# Long-term balancing selection at the *Phosphorus Starvation Tolerance 1* (*PSTOL1*) locus in wild, domesticated and weedy rice (*Oryza*)

**DOI:** 10.1186/s12870-016-0783-7

**Published:** 2016-04-22

**Authors:** Cynthia C. Vigueira, Linda L. Small, Kenneth M. Olsen

**Affiliations:** Department of Biology, High Point University, High Point, NC USA; Department of Biology, Washington University, St. Louis, MO USA

**Keywords:** Balancing selection, Crop domestication, Gene presence/absence variation (PAV), *Phosphorus Starvation Tolerance 1* (*PSTOL1*), Weedy rice, Abiotic stress

## Abstract

**Background:**

The ability to grow in phosphorus-depleted soils is an important trait for rice cultivation in many world regions, especially in the tropics. The *Phosphorus Starvation Tolerance 1* (*PSTOL1*) gene has been identified as underlying the ability of some cultivated rice varieties to grow under low-phosphorus conditions; however, the gene is absent from other varieties. We assessed *PSTOL1* presence/absence in a geographically diverse sample of wild, domesticated and weedy rice and sequenced the gene in samples where it is present.

**Results:**

We find that the presence/absence polymorphism spans cultivated, weedy and wild Asian rice groups. For the subset of samples that carry *PSTOL1*, haplotype sequences suggest long-term selective maintenance of functional alleles, but with repeated evolution of loss-of-function alleles through premature stops and frameshift mutations. The loss-of-function alleles have evolved convergently in multiple rice species and cultivated rice varieties. Greenhouse assessments of plant growth under low- and high-phosphorus conditions did not reveal significant associations with *PSTOL1* genotype variation; however, the striking signature of balancing selection at this locus suggests that further phenotypic characterizations of *PSTOL1* allelic variants is warranted and may be useful for crop improvement.

**Conclusions:**

These findings suggest balancing selection for both functional and non-functional *PSTOL1* alleles that predates and transcends Asian rice domestication, a pattern that may reflect fitness tradeoffs associated with geographical variation in soil phosphorus content.

**Electronic supplementary material:**

The online version of this article (doi:10.1186/s12870-016-0783-7) contains supplementary material, which is available to authorized users.

## Background

Abiotic stresses, such as drought, high salinity and low soil nutrient levels, negatively impact crop production worldwide and are predicted to increase in coming decades due to climate change [1, 2]. Much work has focused on identifying molecular mechanisms of abiotic stress tolerance in plants with the goal of breeding more tolerant crops [3, 4]. However, the genetic basis of plant adaptation to abiotic stress remains poorly understood, even in genomic model species such as rice. Identification of stress tolerance genes is often limited to mutant lines or a relatively few individuals used in mapping populations, and wider analysis of natural allelic variants in cultivated and wild populations is rare (but see [5–7]).

One factor that may shape the evolution of abiotic stress adaptation is fitness trade-offs in contrasting environments, where genotypes that can tolerate abiotic stress show reduced competitive ability or yields when grown in more favorable conditions. At the genetic level, antagonistic pleiotropy describes genotypes that are adaptive in one environment but that negatively impact fitness in another [8, 9]. Fitness trade-offs for stress tolerance may be especially important for crops such as rice (*Oryza sativa)*, where cultivated varieties show tremendous variation in their agroecological adaptations. In rice this range includes upland, drought-tolerant varieties; deep-water varieties that grow in monsoon-flooded fields; short-season, cold-adapted varieties grown at high elevations and latitudes; and varieties with high tolerance to salinity and nutrient-poor soils [10].

Rice is the primary staple food for over one-third of the world’s population [11], and rice cultivation extends over 80 nations spanning six continents [10]. Soil phosphorus (P) availability varies widely across rice production areas. A large proportion of crop varieties are grown in P-depleted soils, particularly in upland regions of tropical Asia [12, 13]. At the same time, rice is also widely cultivated in P-rich or P-supplemented soils, and the varieties grown in these conditions are typically high yielding but intolerant of nutrient deprivation. This pattern suggests potential fitness trade-offs associated with tolerance of nutrient-poor soils. At the genetic level, if spatial heterogeneity in soil P availability has selected for both low-P-tolerant and -intolerant genotypes, then genes underlying this adaptive variation would be expected to evolve under balancing selection to maintain both allele classes. To the extent that soil nutrient heterogeneity also affects populations of rice’s wild and weedy relatives, non-cultivated *Oryza* populations could also be subject to balancing selection for low-P tolerance.

Asian cultivated rice (*O. sativa*) consists of two major subspecies, *japonica* and *indica*, which were domesticated from wild rice, *O. rufipogon,* starting about 8000 years ago [14]. The *indica* subspecies consists of *indica* and *aus* varieties, and the *japonica* subspecies consists of *tropical japonica*, *temperate japonica*, and *aromatic* varieties. All five cultivated variety groups can be distinguished genetically [15]. In addition to domestication, Asian rice has also undergone de-domestication, leading to conspecific weedy strains that infest crop fields. Several genetically distinct weedy rice populations have been described, including straw-hulled U.S. weeds derived from *indica* crop progenitors [16], black-hulled U.S. weeds from *aus* progenitors [16], Korean weeds from *indica* progenitors [17], Korean weeds from *japonica* progenitors [17], and Malaysian weeds from *indica* progenitors [18]. In Africa, an independent domestication event starting about 3500 years ago led to African cultivated rice (*O. glaberrima*), which is derived from the wild African species *Oryza barthii* [19, 20]. Both Asian and African domesticated rice belong to the AA genome *Oryza* species clade, which, in addition to their wild progenitor species, includes the South American species *O. glumaepatula*, the Australian species *O. meridionalis,* and the African species *O. longistaminata* [21].

*Phosphorus Starvation Tolerance 1* (*PSTOL1*) has recently been identified as a major genetic determinant of low-P tolerance in rice [22]. This gene, which encodes a protein kinase conferring P starvation tolerance, was identified in a low-P tolerant *aus* rice cultivar, Kasalath [23]. Interestingly, *PSTOL1* was found to be absent from the rice reference genome (Nipponbare, a *temperate japonica* variety), where it falls within a ~90 kb insertion-deletion polymorphism (indel) on chromosome 12. Prior to the functional characterization of *PSTOL1* by Gamuyao et al., this ~90 kb indel was identified as widely polymorphic across Asian rice varieties; insertions are associated with upland rice grown in poor soil conditions, and deletions are associated with lowland rice grown in favorable soil conditions [13]. Given that the ~90 kb indel likely corresponds to *PSTOL1* presence/absence, this pattern suggests that the *PSTOL1* presence/absence variation could potentially reflect geographically heterogeneous selection for low-P-tolerant and -intolerant genotypes.

Although *PSTOL1* appears to be an important gene underlying adaptation of cultivated rice to poor soil environments, sequence variation at this locus has only been directly examined in a handful of cultivars [22, 24]. In this study we report on *PSTOL1* molecular evolution and distribution of the gene presence/absence polymorphism across wild, cultivated and weedy rice. Using these data we address the following questions: 1) What does the distribution of *PSTOL1* presence/absence variation across cultivated, weedy and wild rice tell us about long-term evolution of adaptation to low-P soil conditions? 2) For plants that carry *PSTOL1*, what does the molecular evolution of this gene reveal about mechanisms of selection on functional variation? 3) How does *PSTOL1* variation correspond to phenotypic variation for low-P tolerance in rice plants grown under controlled greenhouse conditions? Our results suggest long-term balancing selection at the *PSTOL1* locus that predates and transcends rice domestication, with strong and persistent selection to maintain both functional and nonfunctional alleles at this agronomically important gene.

## Results

### Phylogenetic distribution of the *PSTOL1* presence/absence polymorphism

**Fig. 1:**
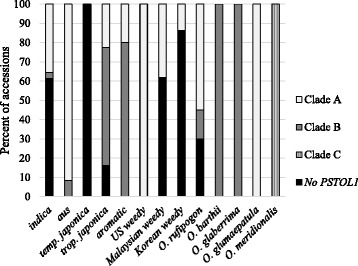
Distribution of *PSTOL1* presence absence polymorphism and clade type. Absence of *PSTOL1* was recorded when no PCR product was produced for any of three sets of primers. Early stop mutations are included in the clade type percentages

Of the 282 screened plants, 160 (56.7 %) carried *PSTOL1* and 122 (43.3 %) were negative for all PCR-genotyping reactions (Fig. 1; Additional file 1: Table S1). Across the six AA genome *Oryza* species sampled, all accessions of *O. barthii*, *O. glaberrima*, *O. glumaepatula* and *O. meridionalis* were found to carry the gene. In contrast, the gene was absent in 30 % of *O. rufipogon* samples and 52 % of *O. sativa* samples. These patterns suggest that the *PSTOL1* presence/absence polymorphism is restricted to Asian cultivated rice and its wild progenitor. Within *O. sativa*, the presence/absence polymorphism spans both the *indica* and *japonica* subspecies. *PSTOL1* was present in all *aus* and *aromatic* crop samples (belonging to the *indica* and *japonica* subspecies, respectively), as well as all US weed accessions (*indica* subspecies), while it was absent from all *temperate japonica* samples. *Indica* and *tropical japonica* crop varieties were polymorphic, as were Malaysian and Korean weeds (which comprise a combination of *indica* and *japonica* strains).

### Additional loss-of-function mutations at *PSTOL1*

**Fig. 2:**
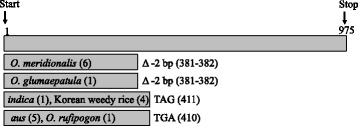
Location of early stop mutations in *PSTOL1* gene. *PSTOL1* consists of a single 975 bp exon. Two different nonsense mutations are located in the same codon (bp 410 and 411). The 410 G to A mutation results in early stop in five *aus* accessions and the 411 G to A mutation results in early stop in one *indica* and four Korean weed accessions. A two basepair deletion at 381 leads to an early stop in a single *O. glumaepatula* accession and six *O. meridionalis accessions*

Although the *PSTOL1* presence/absence polymorphism is restricted to *O. sativa* and *O. rufipogon* in the sampled accessions, the gene appears to have independently evolved loss-of-function alleles across multiple species through other mutational events (Fig. 2). Among the subset of samples that carry the gene, a 2-bp frameshift deletion at nucleotide positions 381–382 was detected in all six *O. meridionalis* accessions; this deletion was also detected in one of the five *O. glumaepatula* accessions. Within *O. sativa*, a G to A substitution at position 411, which results in a premature stop codon at amino acid position 137, was detected in an *indica* crop variety and four Korean *indica-*like weeds. Interestingly, the same codon is altered through an independent premature stop detected in five *aus* varieties and one *O. rufipogon* accession; these samples carry a G to A substitution at nucleotide position 410. Thus, the occurrence of both functional and nonfunctional *PSTOL1* alleles spans multiple AA genome *Oryza* species, and the variation in *PSTOL1* functionality within *O. rufipogon* and *O. sativa* includes both presence/absence variation and loss-of-function mutations. This pattern is consistent with taxonomically- and geographically-widespread selection to maintain polymorphism in *PSTOL1* functionality.

### Evolutionarily diverged *PSTOL1* clades and signatures of balancing selection

**Fig. 3:**
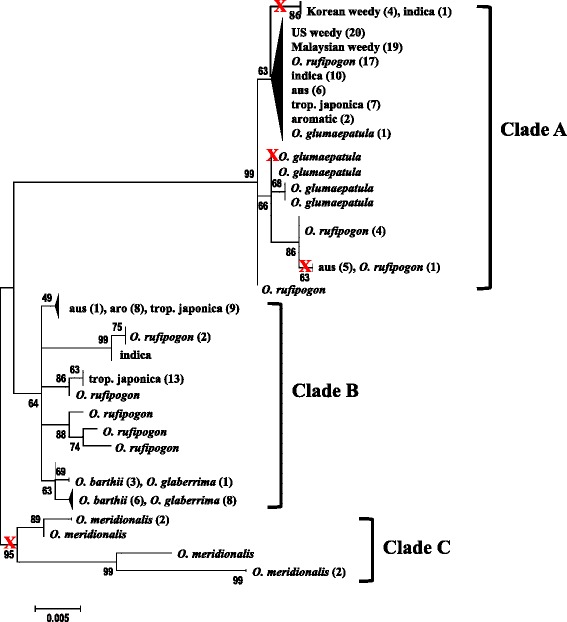
Maximum likelihood phylogeny of *PSTOL1*. Parentheses contain numbers of accessions within a group. Bootstrap values are percentages from 10,000 replicates. The red X symbol indicates locations of early stop mutations

Phylogenetic analysis of *PSTOL1* sequences revealed divergence between *O. meridionalis*, the most distantly related of the sampled AA genome species [21], and the other sampled taxa (Fig. 3, Clade C). For the remaining samples, sequences fell into two well supported and highly diverged clades (Clades A and B), which are separated by 27 mutational steps including 14 nonsynonymous substitutions. Clade distributions among the sampled accessions are shown in Table 1 and in Figs. 1 and 3. Haplotypes of *O. glumaepatula* and African rice (*O. barthii* and *O. glaberrima*) are grouped by species, with *O. glumaepatula* sequences in Clade A and African rice sequences in Clade B (Fig. 3). In contrast, haplotypes of both wild and domesticated Asian rice are present in both clades. Within *O. sativa*, three of the four cultivated variety groups that carry *PSTOL1* are also present in both clades (*indica*, *aus*, *tropical japonica*), with no apparent correlation between clade divergence and *indica/japonica* subspecies designation. Given the deep divergence between the two clades and the incongruence of *O. rufipogon/sativa* haplotypes with known species relationships, these distributions suggest long-term selective maintenance of both the A and B allele classes in Asian rice. Consistent with this inference, frequency spectrum-based tests of neutral equilibrium revealed statistically significant signatures of balancing selection for *tropical japonica* sequences (Tajima’s *D* = 2.43, *P* < 0.001; Fu and Li’s *F*_*S*_ = 2.25, *P* < 0.001), although deviations from neutrality were not significant for *aus*, *aromatic* or *O. rufipogon* samples (Table 1). A statistically significant signature of directional selection was found in *indica* samples (Tajima’s *D* = −2.26, *P* < 0.001; Fu and Li’s *F*_*S*_ = 3.08, *P* < 0.001), likely due to only 1 of 12 accessions possessing the B clade allele.

**Table 1:** Summary statistics of *PSTOL1* nucleotide variation by rice group

*Oryza* group	Clade	No. accessions	No. Segregating Sites	π	θ_W_	Tajima’s D	Fu & Li’s F_S_
*O. sativa*							
*- indica*	A & B	12	38	0.0115	0.0226	−2.2578**	3.0801**
*- aus*	A & B	12	34	0.0196	0.0276	−1.3584	−2.1365*
*- tropical japonica*	A & B	26	34	0.0394	0.0235	2.4304**	2.2530**
*- aromatic*	A & B	10	34	0.0291	0.0315	−0.2841	0.7705
All cultivated	A & B	60	47	0.0451	0.0267	2.3701	1.0783
- US weedy	A	20	0	0	0	—	—
- Malaysian weedy	A	19	0	0	0	—	—
- Korean weedy	A	4	0	0	0	—	—
All weedy	A	43	2	0	0	—	—
*O. rufipogon*	A & B	28	56	0.0397	0.0150	−0.2705	−0.1811
*O. barthii*	B	9	3	0.0011	0.0018	0.0253	0.2023
*O. glaberrima*	B	9	3	0	0	−1.5130*	−1.9338*
*O. meridionalis*	C	6	22	0.0202	-	0.9752	1.0558
*O. glumaepatula*	A	5	25	0.0328	0.0369	−0.9924	−1.0669

Pi and Theta are per base pair at silent sites

10,000 coalescent simulations for significance values

*p-value < 0.05

**p-value < 0.001

It is important to note that BLAST searches using *PSTOL1* sequences from each of the major clades revealed no evidence for additional *PSTOL1* gene copies in published databases, nor did we ever amplify both Clade A and B haplotypes in the same accession. Thus, the A and B clades appear to be highly diverged alleles of the same gene rather than paralogous and/or pseudogenized gene copies. Interestingly, *O. sativa* and *O. rufipogon* sequences with premature stop mutations occur exclusively within Clade A (Fig. 3), which might further point to some difference in functionality between the A and B allele classes.

Comparisons of nucleotide diversity between Asian domesticated rice and its wild progenitor further suggest non-neutral evolution at *PSTOL1*. Whereas neutrally evolving genes across the rice genome show evidence of a domestication bottleneck, with a lower diversity in the crop compared to *O. rufipogon* [25], *PSTOL1* nucleotide diversity is instead higher in cultivated rice (see π and θ_W_ values, Table 1). This pattern suggests that balancing selection has maintained elevated diversity at this locus through the domestication event. Consistent with this finding, we observed a high, albeit non-significant, value for Tajima’s D for all cultivated rice samples pooled (Tajima’s *D* = 2.37, *P* > 0.05). In contrast to *O. sativa*, African domesticated rice (*O. glaberrima*) showed no evidence of balancing selection at *PSTOL1* during domestication; diversity is lower in the crop than its wild progenitor, consistent with neutral evolution during the African rice domestication bottleneck [20].

For weedy rice strains, patterns of nucleotide diversity at *PSTOL1* are consistent with demographic bottlenecks that occurred as these weeds evolved from their domesticated progenitors (e.g., [16, 18]). US weeds are fixed for a single, common Clade A haplotype, which is also present in both *indica* and *aus* cultivated variety groups (the inferred progenitors to US weeds; [16]) (Fig. 3). All Malaysian weeds also share this common clade A haplotype, consistent with their close relationship to *indica* crop varieties [18]. The four *indica*-like Korean weed accessions that carry *PSTOL1* are fixed for a different Clade A haplotype; as with the other weedy rice haplotypes, this haplotype is shared with an *indica* crop accession (Fig. 3).

### Greenhouse growth experiments

**Fig. 4:**
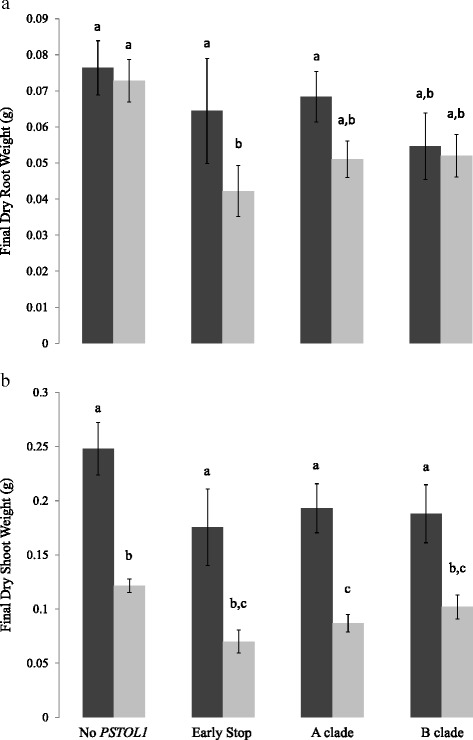
Performance of *PSTOL1* genotypes in low and high phosphorus conditions. Plants were measured after 21 days in high phosphorus (*black*) and low phosphorus (*grey*) media. **a** dry root weight; **b** dry shoot weight

In contrast to the findings of Gamuyao et al. [22], comparisons of genotype performance in low- vs. high-P conditions did not reveal an obvious fitness advantage for plants with functional *PSTOL1* gene copies under low-P conditions. Although plants with functional gene copies showed a non-significant trend towards better growth under low-P conditions than those with premature stop mutations, the plants that performed best of all were those that lack the gene altogether; these results were consistent for both root and shoot growth (Fig. 4a, b, Additional file 2: Figure S2 and Additional file 3: Figure S3). We also found no major difference in root or shoot growth between the two major allele classes (Clades A and B) when plants were grown under low-P conditions. Under high-P conditions, plants grew larger shoots than plants grown in low-P conditions regardless of *PSTOL1* genotype (Fig. 4b and Additional file 3: Figure S3). Root growth did not show significant differences between high- and low-P conditions for plants other than those with premature stop mutations, where root weight was greater under high-P conditions (Fig. 4a and Additional file 2: Figure S2), and for those that lack *PSTOL1,* where roots were longer in low-P conditions (Additional file 4: Figure S1). While our greenhouse experiment was modeled on the methods of Gamuyao et al., our failure to detect the phenotypic associations with *PSTOL1* variation observed in that study may reflect differences in experimental design, including differences in the developmental stages assayed. Two other recent studies have also reported no clear correlation between *PSTOL1* genotypic variation and plant performance in high- vs. low-P conditions [26, 27].

## Discussion

*PSTOL1* is a highly polymorphic gene in AA genome *Oryza* species. We find evidence for the selective maintenance of the gene presence/absence polymorphism across domesticated Asian rice and its wild progenitor (Fig. 1), independent evolution of loss-of-function alleles though nonsense and frameshift mutations (Fig. 2), and maintenance of two distinct clades of functional alleles in Asian rice that bear clear signatures of balancing selection (Fig. 3 and Table 1). The divergence of these two *PSTOL1* clades likely predates Asian rice domestication, as alleles from both clades are found in wild rice (*O. rufipogon*) accessions (Figs. 1 and 3). There is also evidence of long-term balancing selection to maintain the two separate clades across several cultivated rice groups (Table 1). These findings raise intriguing questions on the selective factors that have maintained this functional variation and its implications for adaptation to high- and low-P environments.

### Functional implications of *PSTOL1* variation

There is abundant evidence that *PSTOL1* confers increased uptake of phosphorus in plants that possess functional copies of the gene. This gene, which encodes a receptor-like cytoplasmic kinase [22], was identified through fine mapping of a major QTL for P uptake, *Pup1*, which had been found to explain nearly 80 % of the variation in this trait between Kasalath and the Nipponbare [28]. *PSTOL1* overexpression in two transgenic lines increased grain yield by more than 60 % under low-P conditions, with improved uptake of P and other nutrients arising through inreased root length and surface area [22]. In addition, a survey of 159 cultivated Asian rice accessions indicated that the *Pup1* QTL was differentially represented in varieties adapted to unfavorable, stress-prone growing conditions [13]. Homologs of *PSTOL1* have also been implicated in increased P-uptake efficiency in sorghum [29] and maize [30]. All of these findings would suggest that selection should strongly favor *PSTOL1* functionality rice plants growing in stressful, low-P soil conditions.

Correspondingly, the phylogenetically and geographically widespread occurrence of loss-of function and gene-deletion alleles can most plausibly be explained as an instance of antagonistic pleiotropy, with energetic tradeoffs or other costs disfavoring *PSTOL1* expression in environments with greater P availability. Whereas *PSTOL1* has been completely lost in *temperate japonica* rice, functional copies are present in some *indica, aus, tropical japonica* and African cultivated rices, indicating that phosphorus starvation tolerance could be beneficial in parts of the regions where the latter varieties are cultivated. Transgenic expression experiments provide additional evidence that *PSTOL1* likely has pleiotropic effects. For example, *35S:PSTOL1* overexpression lines cause changes in expression of genes related to root growth and stress response when compared *PSTOL1* null lines [22]; affected genes include those involved in drought response and leaf senescence, two traits that are likely under strong selective constraints of their own. Examples of antagonistic pleiotropy are rare in plants, and further experiments with the *PSTOL1* polymorphism will be required to definitively confirm that functional alleles are selected against in some environmental conditions.

Just as the polymorphism in *PSTOL1* functionality is likely to reflect an adaptive polymorphism, the occurrence of two evolutionarily diverged clades within Asian rice also suggests balancing selection, in this case to maintain two functionally distinct allele classes. Clade A and B haplotypes differ by 14 nonsynonyous subsititutions (4.3 % amino acid divergence), and a subset of these amino acid differences could underlie functional differences in the protein kinase activity. However, the functional differences between Clade A and B alleles, if any, remain to be characterized. The well characterized Kasalath *PSTOL1* allele falls within Clade A haplotypes (Additional file 1: Table S1), and studies to date on the phenotypic effects of *PSTOL1* variation have either focused specifically on that allele [22, 28] or have assessed the *Pup1/PSTOL1* polymorphism without information on underlying sequence variation [13, 26, 27]. Thus, as with the gene presence/absence polymorphism, further work is needed to assess functional differences between the major *PSTOL1* haplotype clades.

Pariasca-Tanaka et al. [24] recently described an African rice *PSTOL1* sequence that matches the Clade B haplotype we found in the majority of our African rice samples (see Fig. 3). They note that while differing at 6 % of amino acid sites from the Kasalath allele, the sequence is conserved in the kinase catalytic domain. Similarly, all but three accessions in our Clade B haplotypes also have a completely conserved kinase catalytic domain. The exceptions include two *O. rufipogon* accessions and one *indica* accession which each contain a single amino acid replacement in that domain. These findings suggest that if there are functional differences in the Clade A and B alleles, they most likely do not arise through alterations of the protein’s catalytic domain.

Given the previously documented evidence that *PSTOL1* enhances P uptake under low-P conditions [13, 22, 28, 31] and our evidence of balancing selection at this locus (Fig. 3 and Table 1), it is somewhat surprising that we did not detect any obvious effects of *PSTOL1* variation on plant growth in the greenhouse (Fig. 4). On the other hand, at least two other recent studies have also found no clear correlation between *PSTOL1* genotypes and plant performance in high- and low-P environments. In a survey of 31 *indica* rice varieties, Sarkar et al. [27] reported that the *Pup1* locus was only present in three of the nine varieties showing greatest efficiency in P uptake in low-and high-P soils. Similarly, Mukherjee et al. [26] found no association between *PSTOL1* presence and P-deficiency tolerance in a study of 108 diverse Indian varieties. The latter study also assessed the effects of *PSTOL1* presence on low-P tolerance using a panel of 180 recombinant inbred lines and again found no evidence that the gene confers increased P-uptake efficiency. Like all physiological responses to abiotic stress, P uptake efficiency is undoubtedly a complex trait, and as such the phenotypic effects of *PSTOL1* would be expected to vary across genetic backgrounds and environments. It seems likely that the variable effects reported for *PSTOL1* in different studies are a manifestation of these gene-by-gene and gene-by-environment interactions.

### Balancing selection

The phylogenetic distribution of putatively functional and non-functional *PSTOL1* alleles suggests that selection has maintained this functional polymorphism across multiple *Oryza* species (Figs. 1 and 3). Trans-specific polymorphisms are typically established when balancing selection has been acting on a gene for very long periods of time [32], indicating that the same differential selective pressures may have been acting in this genus long before the domestication of Asian rice. Examples of trans-specific polymorphism are rare; self-incompatibility loci provide one of the best examples of long term balancing selection in plants (reviewed by [33]). However, the selective pressures acting on self-incompatibility are due to negative frequency dependent selection, where rare alleles have a selective advantage over common alleles. Balancing selection on abiotic stress genes is instead likely due to differential selection based on variable environmental conditions.

One example of long term balancing selection on abiotic stress genes was found in two species of wild tomato *Solanum peruvianum* and *S. chilense* [34]. The C-repeat binding factor (*CBF2*) is involved in plant responses to cold and drought, environmental conditions that vary across *Solanum* species ranges. Mboup et al. found trans-species polymorphism at *CBF2* that maintains signatures of balancing selection. Environmental heterogeneity is likely driving balancing selection in both *CBF2* and *PSTOL1*, and is likely not limited to these two examples of abiotic stress tolerance genes. Genome-wide studies of polymorphism in plants have identified several genomic regions that have signatures of balancing selection (e.g. [35]), but few studies have looked across species for trans-species polymorphisms in closely related plants.

## Conclusions

Agricultural environments exhibit a range of abiotic stresses including drought, low nutrient availability and high salinity. Geographical variation in these stresses can lead to balancing selection at loci that underlie adaptation to these stresses. Our findings for *PSTOL1* suggest that adaptive variation at this gene has been a target of environmentally heterogeneous selection not only within cultivated rice, but also on a larger time frame and geographical scale that encompasses multiple AA genome *Oryza* species. While we did not observe clear phenotypic associations with *PSTOL1* molecular variation in the greenhouse, the molecular signature of balancing selection we detect at this locus suggests that functional variation is important in the field — both in cultivated rice and wild *Oryza* species. It is likely that other genes involved in abiotic stress response will also show signatures of balancing selection in crop plants and their wild relatives.

## Methods

### *PSTOL1* genotyping and sequence analysis

Rice seeds were obtained from germplasm collections of the United States Department of Agriculture (USDA), the International Rice Research Institute (IRRI) or from Dr. Beng Kah Song (Monash University Malaysia), who kindly provided Malaysian weedy rice samples. Plants were grown to the seedling stage (10–20 cm), and young leaf tissue was collected for DNA extraction. Genomic DNA was extracted using Qiagen DNeasy kits or a modified CTAB extraction protocol [36]. DNA quality and quantity were determined using 0.8 % agarose gel electrophoresis and ethidium bromide staining. In total, 282 plants were included in the analysis, representing six AA genome *Oryza* species: *O. barthii* (*N* = 9), *O. glaberrima* (*N* = 9), *O. glumaepatula* (*N* = 5), *O. meridionalis* (*N* = 6), *O. rufipogon* (*N* = 40), and *O. sativa* (*N* = 114 crop varieties, 99 weed accessions) (Additional file 1: Table S1).

The *PSTOL1* presence/absence polymorphism was scored using three sets of PCR primers spanning different portions of the gene, which consists of a single exon of 975 bp (Additional file 5: Table S2). PCR amplifications followed standard protocols [36] and were optimized before large scale screening. Plants that yielded a negative PCR result at least twice for all three primer pairs were scored as absent for the *PSTOL1* gene. If any of the three primer pairs individually yielded a product while the other two primer pairs were negative, the amplicon was sequenced; however, there were no cases where the resulting product had sequence similarity to *PSTOL1*, and these plants were therefore scored as negative. PCR cleanup was carried out using Wizard PCR cleanup kits. All amplicons were direct Sanger sequenced in both directions following previously described protocols [36].

*PSTOL1* sequences were contiged, aligned and checked for quality using Phred/Phrap and BioLign version 4.0.6.2 (Tom Hall, http://en.bio-soft.net/dna/BioLign.html). Only high quality base calls (quality score of 30 or better) were retained. Alignments were trimmed to the start and stop codon for the *PSTOL1* gene. Evidence for heterozygous SNPs (double peaks) was screened by eye and found to be absent in the dataset, consistent with the highly selfing nature of both cultivated and weedy rice.

A Maximum Likelihood tree was constructed using MEGA version 5 [37] with complete deletion of gaps and 10,000 bootstrap replicates. Tree construction employed a GTR plus gamma mutation model, which was selected using Akaike information criteria as implemented in Modeltest [38]. DnaSP version 5 [39] was used to calculate summary statistics for genetic diversity, including numbers of segregating sites, average pairwise nucleotide diversity at silent sites (π) with Jukes-Cantor correction, and Watterson’s estimator of θ at silent sites. Neutral-equilibrium models were tested by estimating Tajima’s D amd Fu’s F_S_ statistics in DnaSP with 10,000 coalescent simulations to assess significance.

### Phenotyping for low-phosphorus tolerance

Sensitivity to low-P growing conditions was measured by root growth in low and high phosphorus conditions in the greenhouse following the protocol of Gamuyao et al. [22], as well as by total shoot mass. Twenty-four accessions spanning the taxonomic sampling of the study were included in the experiment (Additional file 1: Table S1). These included six accessions that lack the *PSTOL1* gene; four that have the gene but carry premature stop mutations that would be expected to render it nonfunctional (see Results); and fourteen with putatively functional gene copies. Seeds were germinated on wet filter paper, and three seedling replicates per accession were assayed for each phosphorus treatment. Gamuyao, et al. transferred seedlings after 3 days of germination; however, our seedlings had not all germinated until day 10. At 10 days after germination, seedlings were transferred into styrofoam trays suspended in Yoshida growth media [40], which was changed out every 3 days. High- and low-P growth conditions were established by making the concentration of NaH_2_PO_4_ in the media either 100 uM or 10 uM. Root length was measured every 3 days, and final dry root and shoot weight was taken after 21 days in growth media.

### Ethics

Not applicable.

### Consent to publish

Not applicable.

### Availability of data and materials

The data sets supporting the results in this article are available in Genbank, KU922566 - KU922725.

## Additional files

Additional file 1: Table S1.
*Oryza* samples used in analyses. (XLSX 21 kb) Additional file 2: Figure S2. Dry Root Weights of *PSTOL1* genotypes grown in low and high phosphorus conditions. Plants were measured after 21 days in high phosphorus (black) and low phosphorus (grey) media. (PDF 75 kb) Additional file 3: Figure S3. Dry Shoot Weights of *PSTOL1* genotypes grown in low and high phosphorus conditions. Plants were measured after 21 days in high phosphorus (black) and low phosphorus (grey) media. (PDF 72 kb) Additional file 4: Figure S1. Root lengths of *PSTOL1* genotypes grown in low and high phosphorus conditions. Plants were measured after 21 days in high phosphorus (black) and low phosphorus (grey) media. (PDF 60 kb) Additional file 5: Table S2.
*PSTOL1* primers used in PCR genotyping and sequencing. (XLSX 8 kb)

## Abbreviations

P: phosphorus
