# Supplementary figures and images for: Long-term balancing selection at the Phosphorus Starvation Tolerance 1 (PSTOL1) locus in wild, domesticated and weedy rice (Oryza)
Source: BMC Plant Biol. 2016 Apr 22;16:101. doi: 10.1186/s12870-016-0783-7 (PMC4840956; doi:10.1186/s12870-016-0783-7)

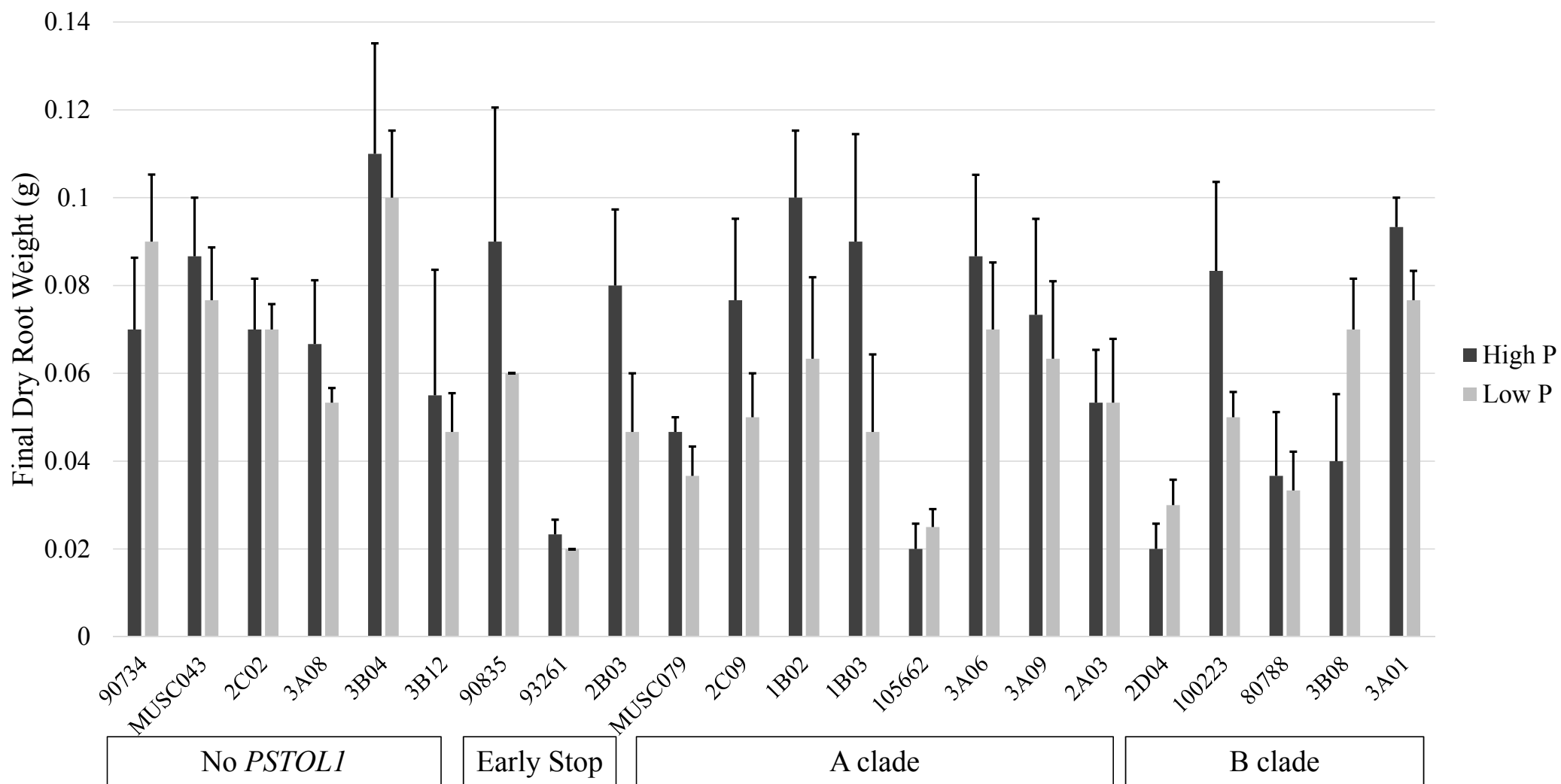

Supplement: Additional file 2: Figure S2. — Dry Root Weights of PSTOL1 genotypes grown in low and high phosphorus conditions. Plants were measured after 21 days in high phosphorus (black) and low phosphorus (grey) media. (PDF 75 kb) [file 12870_2016_783_MOESM2_ESM.pdf]

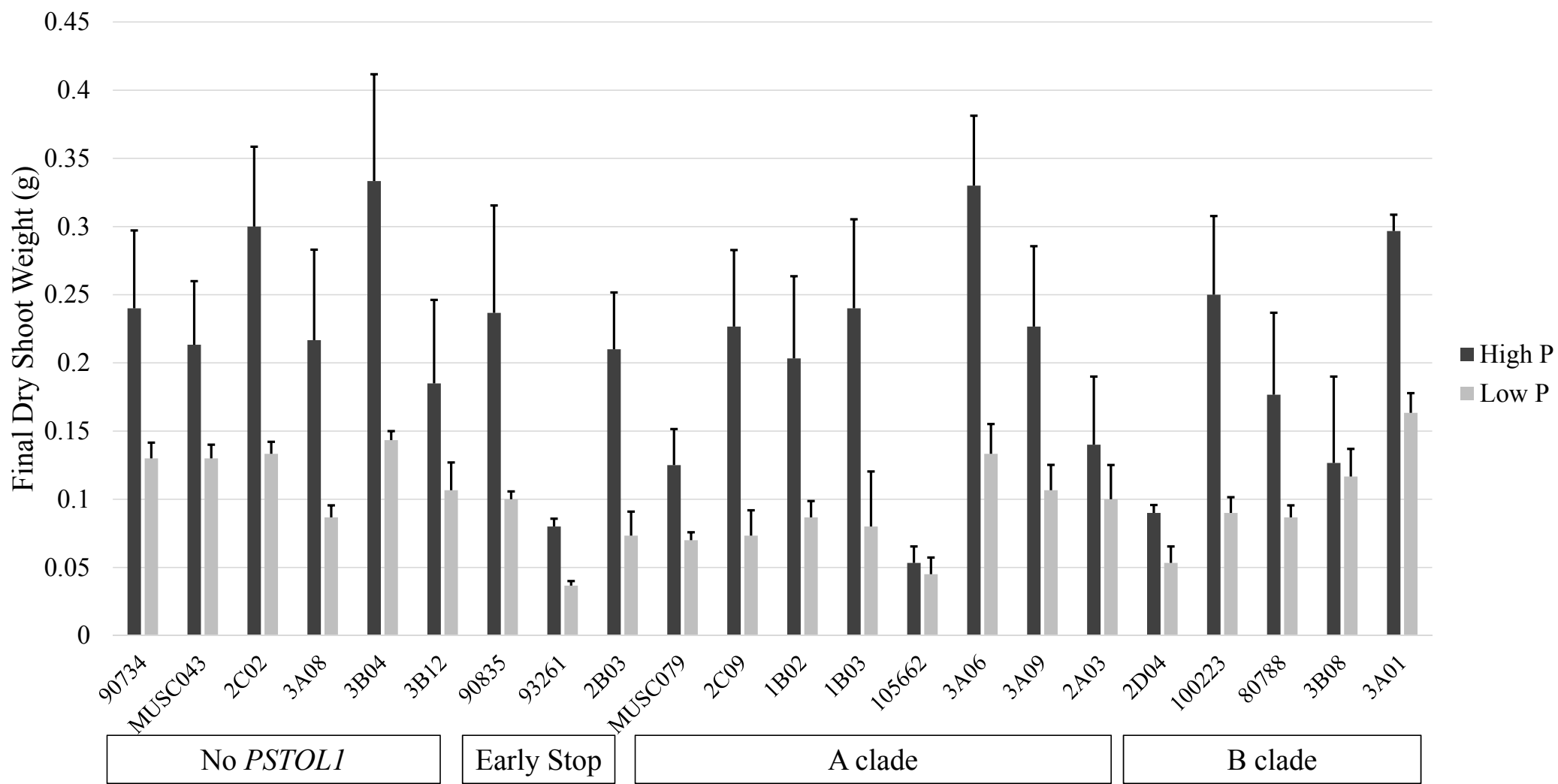

Supplement: Additional file 3: Figure S3. — Dry Shoot Weights of PSTOL1 genotypes grown in low and high phosphorus conditions. Plants were measured after 21 days in high phosphorus (black) and low phosphorus (grey) media. (PDF 72 kb) [file 12870_2016_783_MOESM3_ESM.pdf]

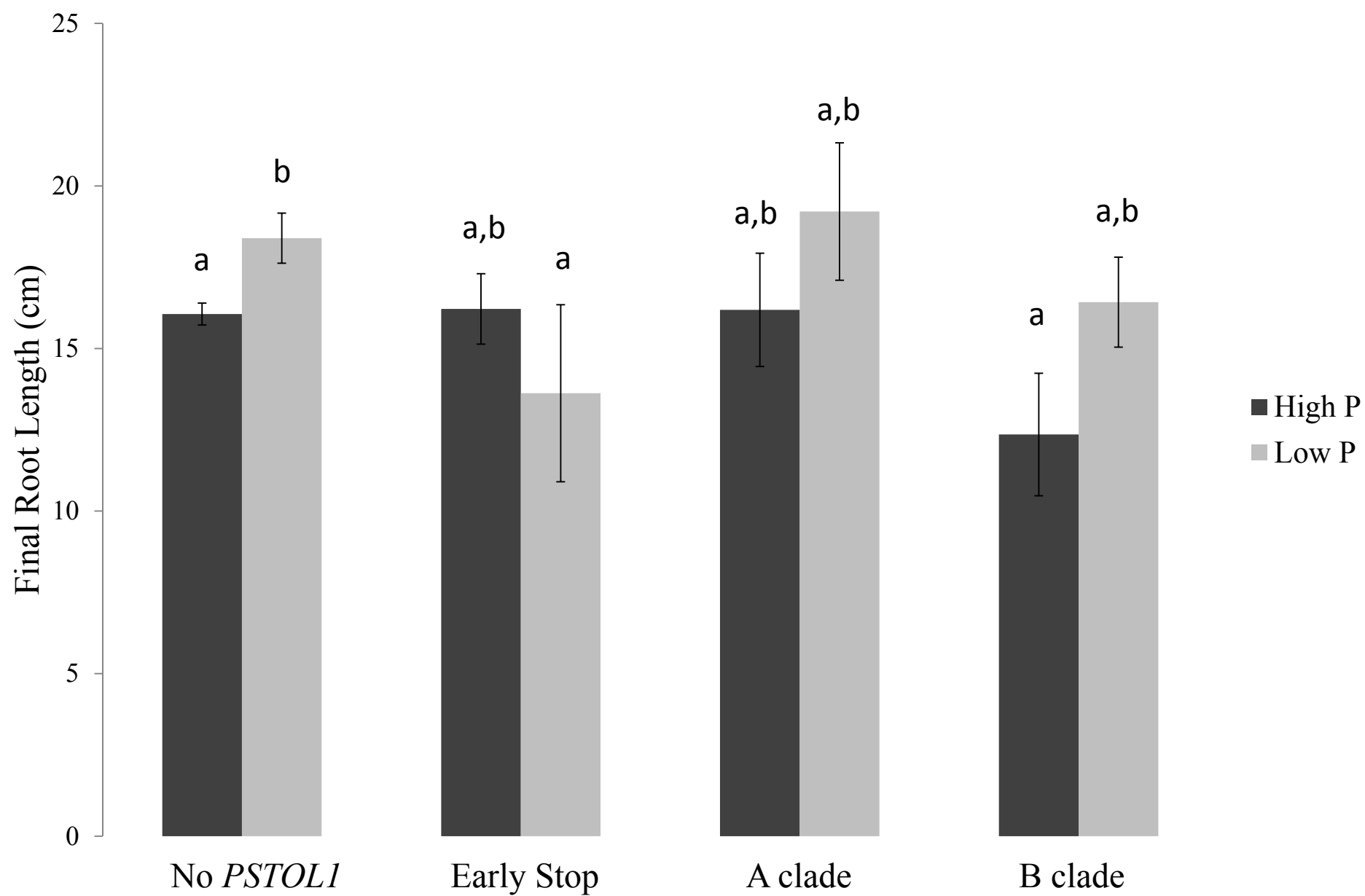

Supplement: Additional file 4: Figure S1. — Root lengths of PSTOL1 genotypes grown in low and high phosphorus conditions. Plants were measured after 21 days in high phosphorus (black) and low phosphorus (grey) media. (PDF 60 kb) [file 12870_2016_783_MOESM4_ESM.pdf]
